# KDH-Net: Explainable Medical AI for Multiclass Kidney Disease Characterization from CT Images

**DOI:** 10.3390/jcm15083165

**Published:** 2026-04-21

**Authors:** Md Serajun Nabi, Su Waddy Tun, Shahaba Alam, Muhammad Kabir Abdullahi, Hasanul Bannah, Istiyak Amin Santo, Arbab Sufyan Wadood, Golam Md Mohiuddin, Zaka Ur Rehman, Hezerul Bin Abdul Karim

**Affiliations:** 1 Faculty of AI and Engineering, Multimedia University, Persiaran Multimedia, Cyberjava 63100, Malaysia; 2 School of Computing and Informatics, Albukhary International University, Alor Setar 05200, Kedah, Malaysia; 3 Faculty of Information Science and Technology, Multimedia University, Jalan Ayer Keroh Lama, Bukit Beruang 75450, Malaysia; 4 Centre for Image and Vision Computing, COE for Artificial Intelligence, Multimedia University, Cyberjaya 63100, Malaysia

**Keywords:** kidney disease classification, computed tomography imaging, hybrid deep learning, model calibration and reliability, explainable artificial intelligence (XAI)

## Abstract

**Background:** Accurate differentiation of kidney diseases such as cysts, tumors, stones, and normal tissue from computed tomography (CT) images remains challenging due to overlapping visual characteristics and variability in data distributions. While deep learning approaches have shown promising results, many existing studies rely on image-level data splitting and focus primarily on accuracy, which may lead to overly optimistic performance and limited clinical reliability. **Methods:** This study proposes KDH-Net (Kidney Disease Hybrid Network), a hybrid deep learning framework for multiclass kidney disease characterization that integrates EfficientNetB0, ResNet50, and MobileNetV2 through feature-level fusion. A two-stage training strategy is adopted to enhance optimization stability. To ensure realistic performance assessment, experiments on the primary dataset are conducted under a patient-level evaluation protocol, eliminating potential data leakage. The framework further incorporates calibration analysis, statistical validation, and explainable artificial intelligence to evaluate prediction reliability and interpretability. **Results:** On the patient-level dataset, KDH-Net achieves an overall accuracy of 0.93 with a macro-average F1-score of 0.91, demonstrating balanced performance across all classes. Confidence analysis indicates meaningful alignment between prediction confidence and correctness, while Grad-CAM visualizations highlight anatomically relevant regions associated with each class. **Conclusions:** The results demonstrate that KDH-Net provides a stable, reliable, and interpretable framework for kidney CT characterization. The proposed system is designed to support clinical decision-making by offering trustworthy predictions under realistic evaluation conditions, rather than replacing clinical expertise.

## 1. Introduction

Kidney diseases constitute a major global health concern, contributing substantially to long-term morbidity and healthcare expenditure. Clinical conditions such as renal cysts, kidney stones, and tumors often present with overlapping radiological characteristics, making accurate differentiation challenging, particularly in early or ambiguous cases [1].

Computed tomography imaging plays a central role in renal diagnosis due to its high spatial resolution and ability to capture detailed anatomical and pathological information [2]. Nevertheless, manual interpretation of kidney CT scans remains time-consuming and subject to inter-observer variability, motivating the development of automated and reliable decision support systems. Deep learning, particularly convolutional neural networks, has shown promising performance in kidney disease characterization from CT images [3,4,5]. However, many existing approaches rely on single-backbone architectures and are often designed for binary classification tasks, which limit their applicability in realistic multiclass clinical settings. Moreover, high classification accuracy alone does not guarantee clinical reliability, as deep models frequently exhibit overconfidence and poor calibration, especially under class imbalance and distributional shifts [6]. In addition, several studies adopt image-level data splitting strategies, which may inadvertently introduce data leakage when images from the same patient appear across training and testing sets, leading to overly optimistic performance estimates. These shortcomings raise concerns regarding trust, interpretability, and safe deployment in clinical practice.

In addition, current AI-based studies often lack comprehensive statistical validation and explainability integration. Although visualization techniques such as Grad-CAM have been introduced in some works, they are frequently presented in isolation without systematic analysis or consistency across disease categories [7]. Furthermore, uncertainty quantification, calibration analysis, and stable evaluation across independent datasets remain underexplored, limiting confidence in the reported performance and generalizability of existing models [8].

### 1.1. Study Aim

The study aims to:

- Develop a hybrid deep learning framework that leverages complementary features from multiple backbones for multiclass kidney disease characterization.
- Evaluate model reliability through calibration, confidence estimation, and statistical validation under a patient-level evaluation protocol.
- Enhance interpretability using explainable AI to provide anatomically meaningful and class-consistent visual explanations.

### 1.2. Contributions

**Figure 1:**
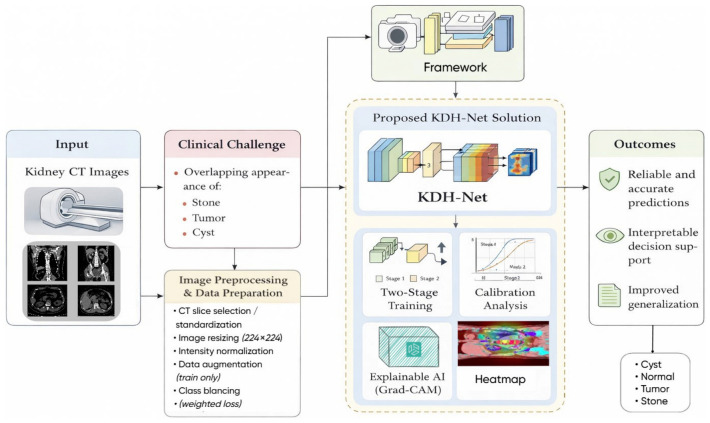
Overview of the proposed KDH-Net framework, illustrating the end-to-end workflow from kidney CT imaging to reliable and explainable multiclass disease characterization.

The study presents KDH-Net, a hybrid multi-backbone framework for multiclass kidney disease classification from CT images. The model integrates EfficientNetB0, ResNet50, and MobileNetV2 through feature-level fusion. A key contribution is the adoption of a patient-level evaluation protocol, ensuring strict data separation and realistic performance assessment. In addition, reliability analysis (calibration and confidence) and Grad-CAM-based explainability are incorporated to support trustworthy and interpretable predictions. Extensive experiments across multiple datasets demonstrate the stable and generalization capability of the proposed framework. The overall workflow of KDH-Net is illustrated in Figure 1.

## 2. Literature Review

Deep learning methods, particularly convolutional neural networks, have been widely applied to kidney disease analysis using CT imaging. Early studies primarily focused on binary characterization tasks, such as distinguishing normal versus abnormal kidneys or detecting the presence of renal tumors, often using single-backbone CNN architectures [3,4,9]. While these approaches demonstrated promising accuracy, their clinical utility remains limited due to the oversimplification of diagnostic categories. More recent works have explored multiclass kidney disease characterization, including differentiation among cysts, stones, tumors, and normal tissue [10,11,12]. These studies highlight the increased complexity of multiclass settings, where class imbalance and inter-class similarity pose significant challenges. Single-network models often struggle to maintain balanced performance across minority classes, leading to degraded macro-level metrics despite high overall accuracy. This limitation motivates architecture capable of capturing richer and more diverse feature representations.

To address the limitations of single-backbone models, ensemble and hybrid CNN architectures have been increasingly investigated in medical image analysis [13,14,15]. Traditional ensemble approaches typically aggregate predictions from multiple independently trained models through voting or averaging. Although such naïve ensembles can improve stability, they often incur high computational costs and fail to exploit complementary feature representations at the representation level. Recent studies emphasize feature-level fusion, where multiple backbone networks operate in parallel and their learned representations are combined before classification [16,17,18]. This strategy enables the model to integrate multi-scale, semantic, and spatial features in a unified embedding space, leading to improved discrimination, particularly in heterogeneous medical datasets. However, many existing hybrid models are evaluated on single datasets or lack rigorous analysis of generalization and reliability, leaving open questions regarding their stability and clinical readiness.

Explainability has become a critical requirement for deploying deep learning systems in clinical environments. Gradient-based visualization techniques such as Class Activation Mapping and Grad-CAM are among the most widely adopted methods for post hoc interpretation in medical imaging [7,19,20,21,22,23]. These approaches generate class-specific activation maps that highlight image regions contributing to model predictions, enabling qualitative assessment of anatomical relevance. Beyond gradient-based methods, model-agnostic techniques such as SHAP have been explored for image-based explanations, although their computational complexity and limited spatial interpretability restrict practical use in high-resolution medical imaging [24]. Recent clinical AI guidelines emphasize that explainability methods should provide anatomically plausible, consistent, and decision-relevant insights, rather than purely visual artifacts [25]. As a result, Grad-CAM remains the most commonly accepted explainability technique in CT-based diagnostic studies.

High classification accuracy alone is insufficient for clinical deployment, as overconfident yet incorrect predictions may pose significant safety risks. Model calibration, which measures the alignment between predicted confidence and true correctness, has therefore gained increasing attention in medical AI research [6,26,27]. Metrics such as expected calibration error (ECE) and reliability curves are commonly used to assess probabilistic reliability. Recent studies demonstrate that deep CNNs are often poorly calibrated despite promising accuracy, particularly in imbalanced and multiclass medical datasets [26]. Confidence-aware evaluation is therefore essential for decision support systems, where predicted probabilities may influence triage, prioritization, or downstream clinical actions. Integrating calibration analysis alongside performance and explainability has emerged as a best practice for evaluating medical AI systems intended for real-world use [27]. A summary of related studies and the distinguishing characteristic of the proposed approach is presented in Table 1.

## 3. Materials and Methods

### 3.1. Dataset

**Figure 2:**
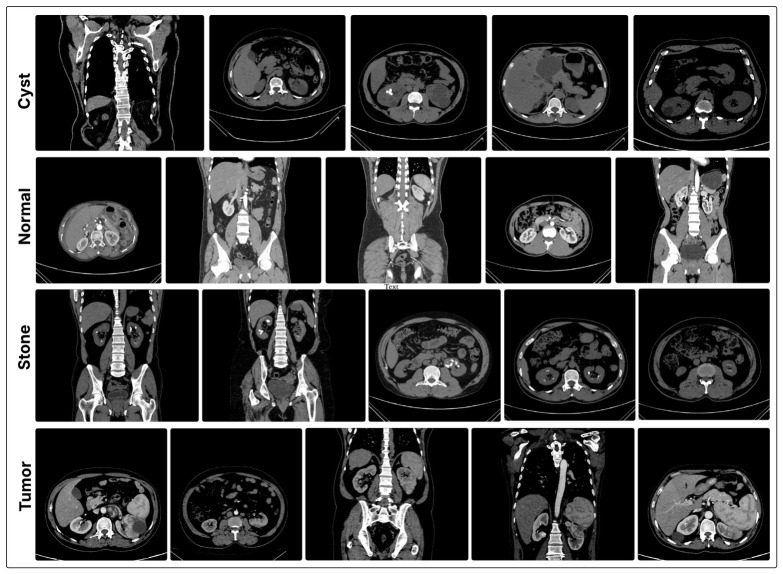
Representative CT image samples from the CT Kidney Dataset illustrating the four diagnostic categories used in this study: Cyst, Normal, Stone, and Tumor.

The experiments were conducted using the publicly available CT Kidney Dataset [35], which contains axial computed tomography (CT) images categorized into four classes: Normal, Cyst, Tumor, and Stone. To ensure a reliable and clinically meaningful evaluation, the dataset was organized at the patient level, where each patient corresponds to a folder (group) containing multiple CT slices. In total, the dataset comprises 217 patient groups distributed across the four classes, with a total of 12,441 CT images. The class-wise distribution of patient groups and corresponding images is summarized in Table 2. Notably, the number of images per patient varies significantly, reflecting real-world clinical acquisition conditions. For model development, a stratified patient-level splitting strategy was adopted to partition the dataset into training, validation, and test sets with approximate ratios of 70%, 15%, and 15%, respectively. All images belonging to a given patient were strictly assigned to a single subset, ensuring that no overlap exists between training, validation, and test sets. This guarantees that the evaluation is performed on entirely unseen patients, thereby preventing data leakage and providing a realistic assessment of model generalization. Representative examples of CT images from each diagnostic category are shown in Figure 2.

All images were resized to a uniform resolution of 224 × 224 pixels and normalized to ensure stable gradient-based optimization. Pixel intensity normalization was performed according to *X*_norm_ = *X*/255, where *X* denotes the original image intensity values. To improve generalization and reduce overfitting, data augmentation techniques, including random rotation, translation, zooming, and horizontal flipping, were applied exclusively to the training set, as summarized in Table 3. To mitigate the impact of class imbalance during learning, a class-weighted loss function was employed, assigning higher penalties to underrepresented classes.

### 3.2. Proposed Hybrid Deep Learning Architecture

The proposed hybrid deep learning architecture, referred to as KDH-Net, is formulated as a multi-branch hybrid convolutional architecture designed to learn complementary representations from kidney CT images through parallel feature extraction and representation-level fusion. Let $X\in R^{H\times W\times 3}$ denote an input CT image resized to a fixed spatial resolution.

**Figure 3:**
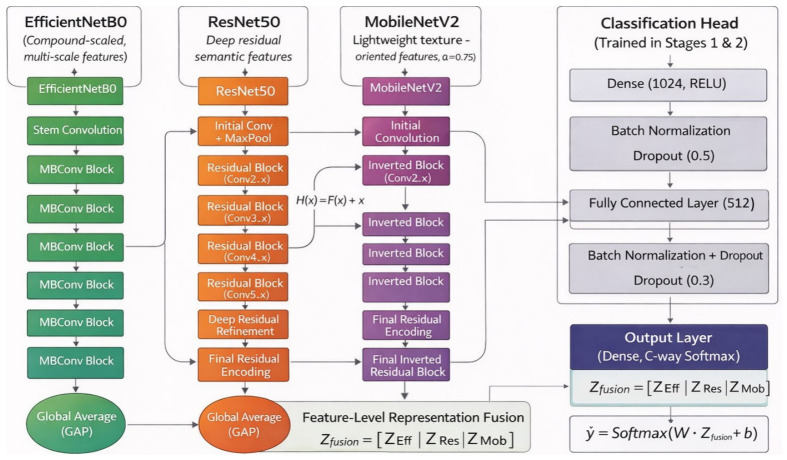
Architecture of proposed KDH-Net: EfficientNetB0, ResNet50, and MobileNetV2 operate as parallel feature extractors. Global average pooling converts backbone feature maps into latent representations, which are fused at the feature level and processed by a regularized classification head.

The architecture consists of three pretrained backbone networks followed by a unified fusion and classification module. The overall architecture is illustrated in Figure 3.

#### 3.2.1. Backbone Networks

Three convolutional backbone networks are employed to extract heterogeneous feature representations from the same input image *X*. Each backbone introduces a distinct inductive bias, enabling the hybrid model to capture diverse spatial and semantic characteristics. The backbone feature extraction process is defined as:

(1) $$F_{Eff}=\Phi_{Eff}(X)\;F_{Res}=\Phi_{Res}(X)\;F_{Mob}=\Phi Mob(X)$$

where Φ_Eff_, Φ_Res_, and Φ_Mob_ denote the nonlinear mappings implemented by EfficientNetB0, ResNet50, and MobileNetV2, respectively, and $F_{i}\in R^{h_{i}\times w_{i}\times c_{i}}$ represents the resulting feature tensor. EfficientNetB0 introduces compound scaling across depth, width, and resolution, allowing balanced multi-scale feature extraction with reduced parameter complexity. ResNet50 employs deep residual learning, governed by the residual transformation. The selection of backbone networks is further supported by empirical analysis presented in Section 5, where individual and hybrid combinations are systematically compared. Although EfficientNetB0 exhibits low standalone performance, its inclusion consistently improves hybrid models, indicating complementary feature contribution. This observation justifies the selection of EfficientNetB0, ResNet50, and MobileNetV2 based on their collective performance rather than individual accuracy.

(2) $$H_{l}=F_{l}\left(H_{l-1}\right)+H_{l-1}\;\;\;\;\;\;$$

which preserves gradient flow across layers and enables stable learning of high-level semantic features. MobileNetV2 utilizes inverted residual blocks and depth wise separable convolutions to emphasize lightweight texture-oriented representations, complementing the deeper semantic abstractions learned by the other backbones.

#### 3.2.2. Feature Fusion Strategy

To transform spatial feature tensors into compact latent representations suitable for joint learning, Global Average Pooling is applied independently to each backbone output. The pooled feature vectors are obtained as:

(3) $$Z_{i}=\frac{1}{h_{i}w_{i}}\sum_{u=1}^{h_{i}}\sum_{v=1}^{w_{i}}F_{i}\left(u,v\right),\;i\in \{\mathrm{Eff},\mathrm{Res},\mathrm{Mob}\}\;\;\;\;\;\;\;\;$$

where $Z_{i}\in R^{c_{i}}$ denotes the channel-wise aggregated representation of the *i*-th backbone. The hybrid representation is constructed through feature-level concatenation

*Z*_fusion_ = [*Z*_Eff_, *Z*_Res_, *Z*_Mob_] (4)

This formulation preserves backbone-specific information while enabling joint optimization over heterogeneous feature spaces. Unlike decision-level fusion, representation-level fusion allows the model to learn cross-backbone feature interactions during training, which constitutes a key contribution of the proposed architecture.

#### 3.2.3. Classification Head

The fused representation *Z*_fusion_ is processed by a fully connected classification head that performs a nonlinear transformation and regularized decision-making. The transformation is defined as:

*H*_1_ = *σ*(BN(*W*_1_*Z*_fusion_ + *b*_1_)) (5)

*H*_2_ = *σ*(BN(*W*_2_*H*_1_ + *b*_2_))

where *W_k_* and *b_k_* denote trainable weight matrices and bias vectors, BN represents batch normalization, and *σ*(·) is the ReLU activation function. Dropout regularization is applied after each hidden layer to reduce overfitting and improve generalization. The final prediction is obtained through a softmax layer.

(6) $$\hat{y}=\mathrm{Softmax}\left(W_{o}H_{2}+b_{o}\right)\;\;\;\;\;\;\;\;\;\;\;$$

where $\hat{y}\in R^{C}$ represents the predicted class probability distribution over *C* disease categories.

### 3.3. Training Strategy

KDH-Net was optimized using a structured two-stage training strategy designed to separate task-specific decision learning from domain-specific representation adaptation. The complete optimization procedure, parameter states, and hyperparameter settings are summarized in Table 4. A brief interpretation of the training stages is provided below for clarity.

In the first stage, all backbone networks were kept fixed, and only the classification head was optimized, allowing stable alignment between pretrained representations and the target task. In the second stage, selective fine-tuning was applied by unfreezing the final layers of each backbone network to enable controlled domain adaptation at higher semantic levels while preserving low-level feature invariance. Class imbalance was addressed consistently across both stages through weighted loss optimization.

The overall architecture construction, two-stage training procedure, and post-training analysis of KDH-Net are summarized in Algorithm 1. The algorithm provides a compact, stepwise representation of the parallel feature extraction and fusion strategy. The experiments were conducted under the computational environment detailed in Table 5.
**Algorithm 1** Two-Stage Training Procedure for KDH-Net**Require:** Labeled dataset $D=\{\left(x_{i},y_{i}\right){\}}_{i=1}^{N}$, pretrained backbones *B* = {Eff, Res, Mob},  learning rates *η*_1_, *η*_2_, class weights *w*, epochs *N*_1_, *N*_2_ **Ensure:** Optimized hybrid model M*   1: Preprocess $x_{i}\to \tilde{x_{i}}\in R^{224\times 224\times 3}$
  2: Initialize backbones *B* with ImageNet weights   3: Parallel feature extraction: *F_b_* = *f_b_*($\tilde{x}$), *b* ∈ *B*   4: Global Average Pooling: *z_b_* = *GAP*(*F_b_*)   5: Feature fusion: $z=\left[z_{\mathrm{Eff}}∥z_{\mathrm{Res}}∥z_{\mathrm{Mob}}\right]$
  6: Classification head: $\hat{y}$ = Softmax(*g*(*z*))   **Stage 1: Frozen Backbone Training**   7: Freeze *θ_B_*, train *θ*_head_   8: **for** epoch = 1 to *N*_1_ **do**   9:    **for** each (*x*, *y*) ~ *D*
**do**
  10:     Compute loss: *L* = −∑*_c_ w_c_ y_c_* log($\hat{y}$*_c_*)   11:     Update head parameters: *θ*_head_ ← *θ*_head_ − *η*_1_∇*L*   12:   **end for**   13: **end for**   **Stage 2: Fine-Tuning**   14: Unfreeze last layers *θ_B_L*, freeze BN layers   15: **for** epoch = 1 to *N*_2_ **do**   16:    **for** each (*x*, *y*) ~ *D*
**do**   17:       Compute loss: *L*   18:       Update parameters: {*θ*_head_, *θ_B_L*} ← {·} − *η*_2_∇*L*   19:    **end for**   20: **end for**   21: **return**
M*

### 3.4. Evaluation Metrics

Model performance was evaluated using complementary metrics that capture correctness, class balance sensitivity, statistical agreement, and threshold-independent discrimination. Metrics derived from the confusion matrix were used to quantify overall and class-wise predictive accuracy. Agreement-based metrics were employed to assess reliability beyond chance, which is critical in medical image analysis. Curve-based metrics were used to evaluate discriminative performance across decision thresholds, with particular emphasis on minority-class behavior. To ensure stable validation, the proposed model was evaluated using a held-out test set and further assessed across multiple independent external datasets. This approach provides a stronger estimate of generalization than conventional k-fold cross-validation, as it evaluates performance under real-world distributional shifts.

Let *TP*, *FP*, *TN*, and *FN* denote the elements of the confusion matrix. Overall classification performance is measured using accuracy

(7) $$\mathrm{Accuracy}=\frac{TP+TN}{TP+TN+FP+FN}\;\;\;\;\;\;\;\;\;\;\;$$

To account for class imbalance, class-wise performance is evaluated using the F1 score

(8) $$F1_{c}=\frac{2\cdot TP_{c}}{2\cdot TP_{c}+FP_{c}+FN_{c}}\;\;\;\;\;\;\;\;$$

To quantify prediction reliability beyond chance agreement, Cohen’s Kappa is used

(9) $$\kappa =\frac{p_{o}-p_{e}}{1-p_{e}}\;\;\;\;\;\;\;\;\;\;\;\;$$

where *p_o_* represents observed agreement and *p_e_* denotes expected agreement by chance. In addition, the Matthews Correlation Coefficient is employed as a balanced correlation measure that incorporates all confusion matrix elements

(10) $$\mathrm{MCC}=\frac{TP\cdot TN-FP\cdot FN}{\sqrt{\left(TP+FP\right)\left(TP+FN\right)\left(TN+FP\right)\left(TN+FN\right)}}\;\;\;\;\;\;\;$$

These metrics provide robust evaluation under class imbalance and asymmetric error distributions.

To evaluate the reliability of probabilistic predictions, calibration analysis was incorporated into the evaluation framework. Model calibration was assessed using reliability curves, which compare predicted confidence levels with observed accuracies across probability bins. To summarize this relationship quantitatively, expected calibration error (ECE) was computed as a scalar measure that captures the average deviation between model confidence and empirical accuracy across bins. Formally, ECE is defined as

(11) $$\mathrm{ECE}=\sum_{m=1}^{M}\frac{\left|B_{m}\right|}{N}\left|\mathrm{acc}\left(B_{m}\right)-\mathrm{conf}\left(B_{m}\right)\right|\;\;\;\;\;\;\;\;\;\;\;\;\;\;$$

where *B_m_* denotes the set of samples whose predicted confidence falls within the *m*-th bin, acc(*B_m_*) represents the empirical accuracy within that bin, and conf(*B_m_*) denotes the mean predicted confidence. In addition, prediction confidence distributions were analyzed to examine whether correct predictions are associated with higher confidence scores than incorrect ones. This analysis enables assessment of confidence reliability, which is essential for clinical decision support systems.

### 3.5. Explainable Artificial Intelligence (XAI) Framework

To provide interpretability at the decision level, KDH-Net was integrated with a gradient-weighted class activation mapping framework. Grad-CAM was applied to the final convolutional layer of the network to generate class-specific activation maps. The class-specific importance of spatial features is computed by weighting the convolutional feature maps according to their contribution to the predicted class score. The Grad-CAM activation for class *c* is computed as

(12) $$L_{\mathrm{GradCAM}}^{c}=\mathrm{ReLU}\left(\sum_{k}\alpha_{k}^{c}A^{k}\right)\;\;\;\;\;\;\;\;$$

where *A_k_* denotes the *k*-th feature map of the selected convolutional layer *k* and *α^c^* represents its corresponding importance weight for class *c*, obtained from gradient information. These maps highlight spatial regions that contribute most strongly to the model’s predictions. The same visualization protocol was applied across all disease categories to ensure consistent and comparable interpretability. This framework allows qualitative inspection of whether the model attends to anatomically meaningful regions during classification.

**Figure 4:**
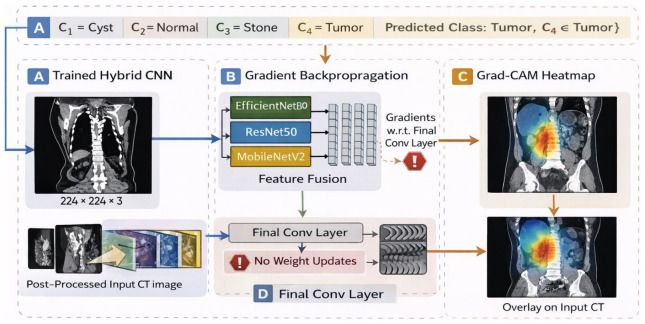
Post hoc Grad-CAM integration with the trained KDH-Net illustrating class-specific attention regions for visual explanation. (**A**) Input CT image and predicted class, (**B**) gradient backpropagation through the hybrid backbone (EfficientNetB0, ResNet50, MobileNetV2), (**C**) generation of Grad-CAM heatmap, and (**D**) projection of the heatmap onto the input image. Arrows indicate the data flow, while highlighted symbols denote gradient computation and feature extraction steps.

The post hoc integration of Grad-CAM with the trained KDH-Net, including gradient backpropagation to the final convolutional layer and heatmap overlay on the input image, is illustrated in Figure 4.

To quantitatively evaluate the reliability of Grad-CAM explanations, perturbation-based metrics were employed. Specifically, confidence drop, deletion, insertion, and masked-region confidence were computed by progressively modifying input images based on Grad-CAM importance maps. These metrics assess whether regions identified as important by the model meaningfully influence prediction confidence.

## 4. Results

The quantitative performance of KDH-Net was evaluated on the held-out test set comprising 1919 CT images from a group of 217 patients. Overall performance indicates promising generalization, with high predictive accuracy and balanced behavior across classes. The model achieved a test accuracy of 0.93, demonstrating reliable discrimination among the four kidney conditions. Both macro-averaged and weighted performance scores remained consistently high, indicating that classification performance was not dominated by majority classes despite the inherent class imbalance.

Under this setting, the proposed KDH-Net achieved an overall accuracy of 0.93, as summarized in Table 6. The results demonstrate that the model maintains promising discriminative capability across all classes, with particularly high performance in the Cyst and Normal categories. A slight performance reduction is observed for tumor cases, which can be attributed to increased inter-patient variability and class imbalance. Overall, this experiment confirms that the proposed framework does not rely on data leakage and remains stable under clinically realistic evaluation conditions, thereby strengthening the validity of the reported results.

### 4.1. Class-Wise Performance and Training Behavior Analysis

**Figure 5:**
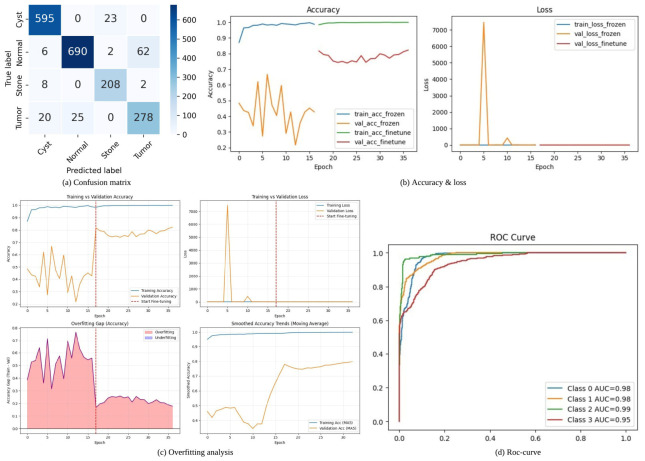
Class-wise performance analysis of the proposed model: (**a**) confusion matrix, (**b**) training and validation accuracy and loss, (**c**) overfitting and convergence analysis (the vertical dotted line marks the start of the fine-tuning phase), and (**d**) AUC-Roc curve for all classes.

Figure 5 presents the performance analysis of KDH-Net under patient-level evaluation. The confusion matrix Figure 5a shows promising diagonal dominance across all classes, indicating effective discrimination, although minor confusion is observed between Normal and Tumor cases. The training curves Figure 5b demonstrate rapid convergence with near-perfect training accuracy, while validation accuracy stabilizes at a lower level, reflecting a realistic generalization gap. The overfitting analysis Figure 5c further confirms this behavior, where the gap between training and validation accuracy reduces after fine-tuning, indicating improved model stability. Finally, the ROC curves Figure 5d show high discriminative capability across all classes, with AUC values exceeding 0.95. This confirms that the model maintains promising class separability, even under patient-level constraints.

### 4.2. Calibration Analysis

**Figure 6:**
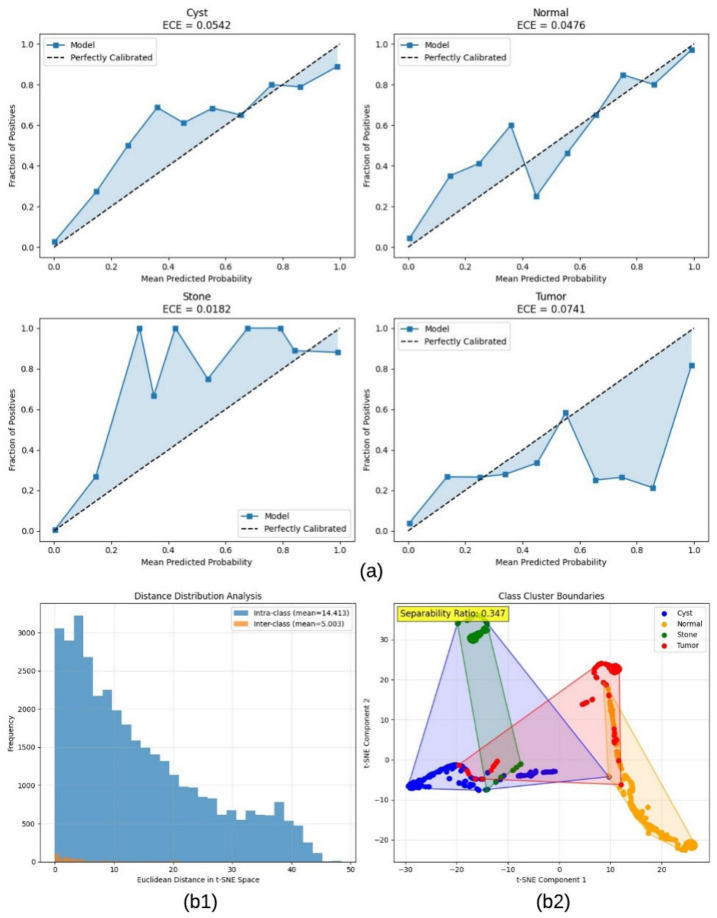
(**a**) Class-wise reliability diagrams show the relationship between predicted confidence and empirical accuracy, with corresponding Expected Calibration Error (ECE) values for each class. (**b**) t-SNE-based analysis of feature space separability. (**b1**) Distribution of intra-class and inter-class Euclidean distances, and (**b2**) two-dimensional projection of feature embeddings illustrating class-wise clustering behavior.

The calibration results in Figure 6a indicate that the model predictions are generally well-aligned with true outcome frequencies, as reflected by relatively low ECE values across all classes. Among them, the Stone class exhibits the best calibration (ECE = 0.0182), while Tumor shows comparatively higher deviation (ECE = 0.0741), suggesting less reliable confidence estimation for more complex or heterogeneous patterns.

The separability analysis in Figure 6b reveals moderate class discrimination in the learned feature space. As shown in Figure 6(b1), the mean intra-class distance (14.41) exceeds the mean inter-class distance (5.00), resulting in a separability ratio of 0.347. This indicates partial overlap between class distributions. The t-SNE visualization in Figure 6(b2) further supports this observation, where clusters are distinguishable but not fully separated, particularly between certain classes.

**Figure 7:**
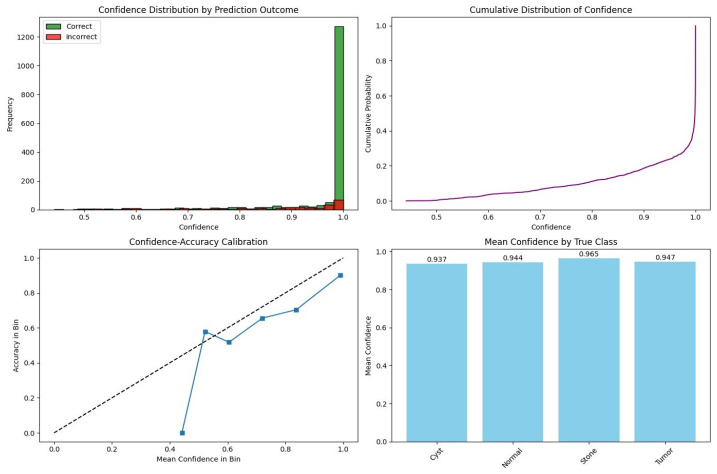
Confidence-based statistical analysis of prediction reliability under patient-level evaluation. Distribution of prediction confidence for correct and incorrect samples. Cumulative confidence distribution. Confidence–accuracy calibration curve and mean prediction confidence across classes.

To further assess prediction reliability, a confidence-based statistical analysis was conducted, as illustrated in Figure 7. The distribution of the prediction confidence plot shows that correctly classified samples are concentrated at higher confidence values, whereas incorrect predictions are more dispersed toward lower confidence regions. This trend is also reflected in the cumulative distribution plot, where predictions are predominantly located in the high-confidence range. The confidence–accuracy calibration curve indicates that the model is generally well-calibrated at higher confidence levels, with minor deviations observed in intermediate regions. Furthermore, the class-wise mean confidence plot remains consistently high across all categories, indicating stable confidence estimation.

To validate this behavior statistically, a Welch’s *t*-test was performed between correct and incorrect predictions. The results show that correctly classified samples exhibit higher confidence (mean = 0.9599) compared to misclassified samples (mean = 0.8544), with an overall mean confidence of 0.9448 and a confidence gap of 0.1055. The test yields a *t*-statistic of 11.1468 with a *p*-value approaching zero, confirming a statistically significant difference. These findings demonstrate that the model’s confidence estimates are strongly aligned with prediction correctness under patient-level evaluation.

#### 4.2.1. Statistical Stability Analysis

**Figure 8:**
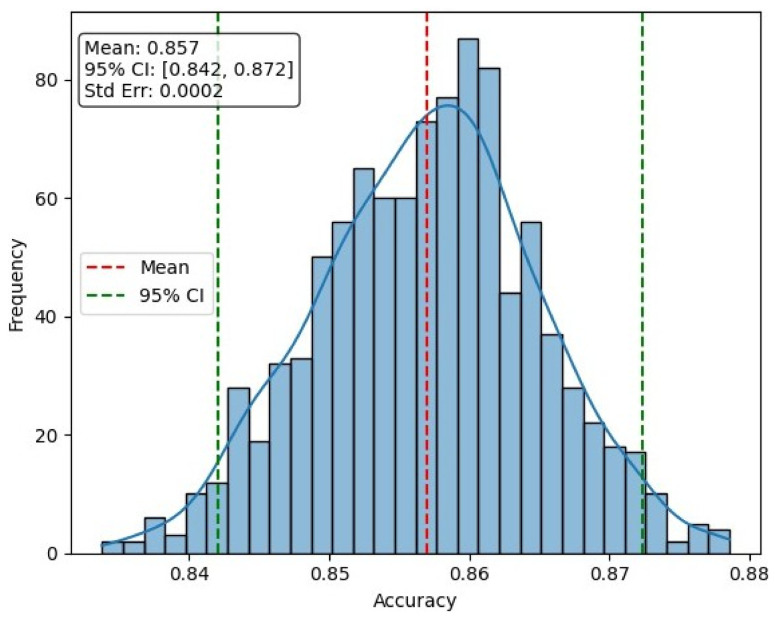
Bootstrap distribution of model accuracy under patient-level evaluation (*n* = 1000), showing mean performance and corresponding confidence intervals.

Model stability was evaluated using bootstrap resampling on the test set, as illustrated in Figure 8 and in Table 7. The bootstrap distribution of accuracy shows a concentrated spread around a mean accuracy of 0.8570, indicating consistent performance across resampled subsets. The 95% confidence interval ranges from 0.842 to 0.872, while the 99% confidence interval spans from 0.837 to 0.877, demonstrating limited variability in model performance. The small standard error (0.0002) further confirms the statistical reliability and stability of the proposed model under patient-level evaluation. Overall, these results indicate that the model maintains stable and reliable performance, with minimal sensitivity to variations in the test data distribution.

#### 4.2.2. Prediction Reliability and Feature Space Analysis

**Figure 9:**
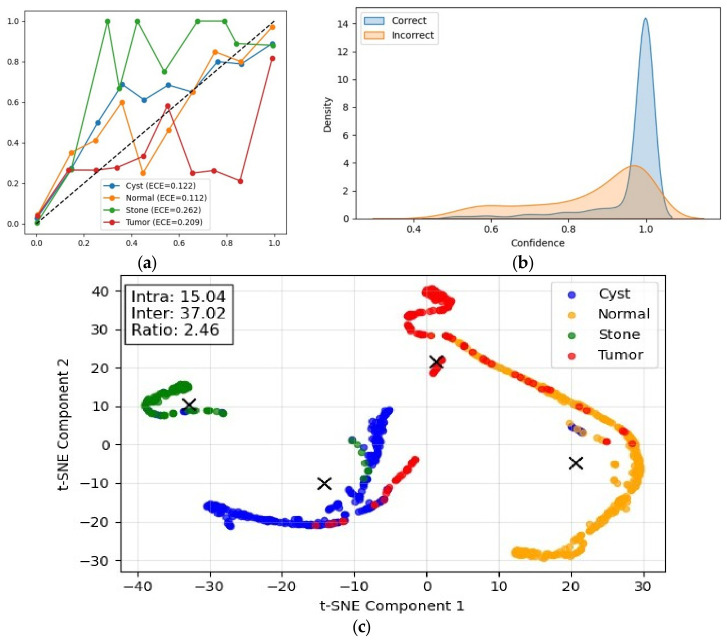
Comprehensive evaluation of prediction reliability and feature representation. (**a**) Class-wise calibration curves with Expected Calibration Error (ECE); the dotted diagonal line represents perfect calibration. (**b**) Confidence distribution for correct and incorrect predictions, illustrating separation between reliable and unreliable predictions. (**c**) t-SNE visualization of feature embeddings showing class separability; colored points denote different classes, while black crosses indicate class centroids. Partial overlap between clusters reflects inherent similarity between certain disease categories but does not significantly affect overall class discrimination.

The reliability of model predictions and the structure of the learned feature space were further analyzed, as shown in Figure 9. The calibration curves in Figure 9a indicate varying levels of confidence alignment across classes. The Normal and Cyst classes exhibit better calibration (ECE = 0.112 and 0.122). In contrast, higher calibration errors are observed for Stone (ECE = 0.262) and Tumor (ECE = 0.209), suggesting less reliable confidence estimates for more complex patterns. The feature space visualization using t-SNE in Figure 9c shows well-formed and distinguishable clusters across all classes. This observation is quantitatively supported by the separability analysis, where the mean inter-class distance (37.02) is substantially larger than the mean intra-class distance (15.04), resulting in a separability ratio of 2.46. This indicates promising class discrimination in the learned representation space. The confidence separation analysis in Figure 9b further demonstrates that correctly classified samples are associated with higher confidence values compared to incorrect predictions. This behavior confirms that the model’s confidence estimates are meaningfully aligned with prediction correctness, supporting its reliability in decision-making under patient-level evaluation.

In addition, a comprehensive set of evaluation metrics is reported in Table 8. The results show low error rates, as reflected by Hamming and zero-one losses (0.1433), along with promising agreement-based performance indicated by the Matthews correlation coefficient (0.7964) and Cohen’s Kappa (0.7959). The balanced accuracy of 0.8496 further confirms consistent performance across classes despite dataset imbalance. Moreover, the overall expected calibration error (ECE = 0.1568) and confidence gap (0.1055) support the reliability of the model’s probabilistic predictions.

### 4.3. Explainability Results

**Figure 10:**
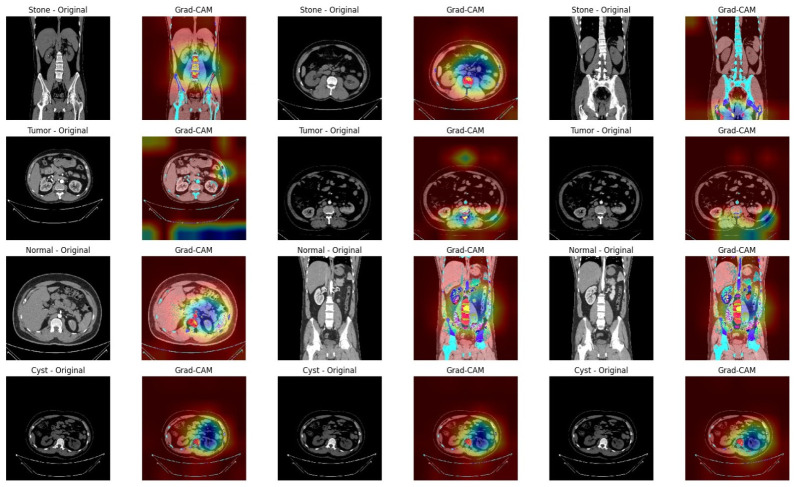
Post hoc Grad-CAM visualizations highlighting class-specific spatial attention patterns learned by KDH-Net across different kidney disease categories. Warmer colors (red/yellow) indicate regions with higher contribution to the model’s prediction, while cooler colors (blue) represent lower relevance.

To enhance the transparency of KDH-Net, post hoc explainability analysis was conducted using gradient-weighted Class Activation Mapping. Representative Grad-CAM visualizations for all classes are shown in Figure 10, where heatmaps are overlaid on the corresponding CT images to highlight the regions that contribute most to the model’s predictions. Across all diagnostic categories, the Grad-CAM activations are predominantly localized within anatomically plausible renal regions. The model consistently focuses on kidney structures while exhibiting minimal attention to surrounding background tissues, such as bowel regions or image margins. This spatial alignment indicates that the learned representations are driven by kidney-specific features rather than spurious correlations, despite the absence of explicit localization supervision during training. Distinct attention patterns are observed across different classes. Normal cases exhibit diffuse and symmetric activation across renal anatomy, without pronounced focal hotspots, suggesting reliance on global structural consistency. Cyst predictions exhibit relatively smooth, homogeneous attention regions, reflecting the uniform appearance of cystic formations. Stone cases are characterized by compact, highly localized activation, consistent with the focal, high-contrast nature of calcified structures in CT images. In contrast, tumor predictions show broader and more heterogeneous attention patterns, indicating reliance on spatially extended regions of altered tissue appearance.

To provide quantitative validation of the Grad-CAM explanations, perturbation-based metrics were evaluated on a representative subset of 200 test images. As summarized in Table 9, the model exhibits an average confidence drop of 0.2913 when important regions are removed, indicating that the highlighted regions contribute substantially to prediction decisions. Furthermore, the confidence retained within the masked important regions (0.4639) demonstrates that Grad-CAM successfully captures discriminative areas relevant to classification. The deletion score (0.5936) and insertion score (0.4247) further confirm that progressively removing important pixels significantly degrades model confidence, while gradually introducing them restores predictive strength. These results provide quantitative evidence that the Grad-CAM explanations are meaningful, consistent, and aligned with the model’s decision-making process, thereby strengthening the interpretability of the proposed KDH-Net framework.

## 5. Discussion

The experimental results demonstrate that KDH-Net achieves consistently high performance across accuracy, stability, calibration, and separability analyses, indicating that the architectural and training choices jointly contribute to effective and reliable classification. The promising test performance, together with stable training behavior, suggests that the model generalizes well beyond the training data rather than relying on dataset-specific cues. The hybrid architecture outperforms single-backbone designs primarily due to complementary feature learning. EfficientNetB0 contributes parameter-efficient multi-scale representations, ResNet50 captures deeper semantic structures through residual learning, and MobileNetV2 emphasizes lightweight spatial feature extraction. By integrating these heterogeneous representations through parallel feature fusion, the model benefits from a richer and more diverse feature space than any individual backbone can provide. This complementary behavior is reflected in the clear class separation observed in the prediction space and the balanced class-wise performance.

The two-stage training strategy further reinforces this effect. Freezing the backbone networks during initial training stabilizes optimization and allows the classification head to adapt effectively to fused features, while subsequent fine-tuning enables selective refinement of high-level representations without disrupting previously learned structures. The minimal gap between training and validation accuracy and the smooth convergence patterns indicate that this strategy mitigates overfitting while preserving discriminative capacity. Class-wise analysis supports these interpretations. Near-perfect precision–recall behavior across all categories demonstrates that the hybrid representation does not favor dominant classes, despite underlying data imbalance. The promising separability observed in t-SNE visualization and distance-based metrics confirms that the model learns structured and well-separated decision regions, rather than relying on marginal boundary adjustments. Moreover, the consistently low calibration error indicates that improved accuracy is accompanied by reliable confidence estimation, which is critical for downstream clinical use.

### 5.1. Comparison with Baseline Models

**Figure 11:**
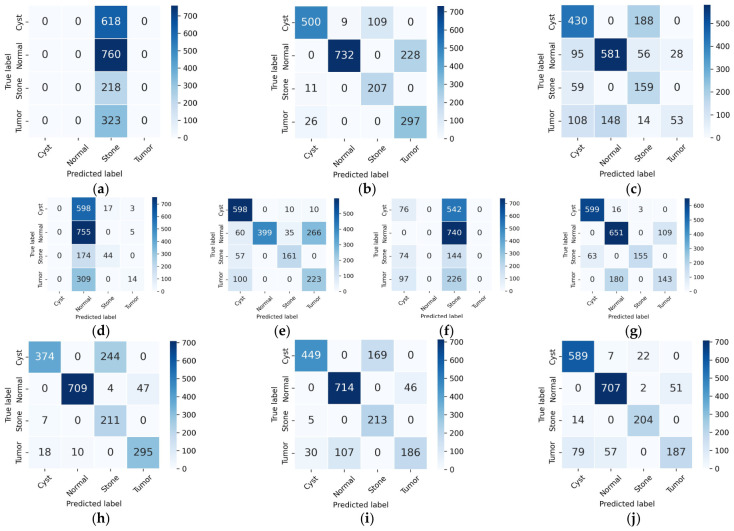
Confusion matrices of baseline models: (**a**) EfficientNetB0, (**b**) InceptionV3, (**c**) MobileNetV2, (**d**) NASNetMobile, (**e**) ResNet50, (**f**) VGG16, (**g**) Xception, (**h**) MobileNetV2 + EfficientNetB0, (**i**) ResNet50 + EfficientNetB0, and (**j**) ResNet50 + MobileNetV2.

A systematic comparative analysis of individual backbone networks and their ensemble combinations was conducted to provide a rigorous justification for model selection. A quantitative summary is presented in Table 10, with corresponding confusion matrices illustrated in Figure 11. All models were trained under identical optimization settings using patient-level data to ensure a fair and leakage-free comparison.

Among individual models, DenseNet121 achieved the strongest performance (accuracy = 0.8828, macro F1 = 0.8495), followed by Xception (accuracy = 0.8067) and InceptionV3 (accuracy = 0.8004). In contrast, EfficientNetB0 exhibited extremely poor standalone performance (accuracy = 0.1136, macro F1 = 0.0510), with predictions collapsing toward a dominant class, as also evident in its confusion matrix in Figure 11. The ResNet50 + MobileNetV2 combination achieved a substantial improvement over individual models (accuracy = 0.8791, macro F1 = 0.8528), approaching the performance of DenseNet121. Likewise, MobileNetV2 + EfficientNetB0 and ResNet50 + EfficientNetB0 achieved accuracies of 0.8280 and 0.8140, respectively. These results indicate that combining models consistently improves performance compared to their individual counterparts. Importantly, although EfficientNetB0 performs poorly as a standalone model, its inclusion in hybrid architecture leads to consistent performance gains. For instance, MobileNetV2 improves from 0.6373 to 0.8280 accuracy when combined with EfficientNetB0. This behavior suggests that EfficientNetB0 contributes complementary feature representations that are not captured by other architectures. A similar trend is observed in the ResNet50 + EfficientNetB0 combination, further supporting this observation.

The confusion matrices in Figure 11 provide additional insight into these behaviors. Single models exhibit significant off-diagonal misclassifications, indicating class bias and inconsistent feature representation. In contrast, hybrid models reduce these misclassifications, and the proposed KDH-Net demonstrates the strongest diagonal concentration, reflecting improved class separability and reduced inter-class confusion. Based on these observations, the selection of backbone networks in KDH-Net is guided by their empirically observed complementary behavior rather than individual performance alone. By integrating ResNet50, MobileNetV2, and EfficientNetB0, KDH-Net leverages diverse feature representations, resulting in the best overall performance (accuracy = 0.93, macro F1 = 0.91) and more balanced class-wise predictions across all categories.

### 5.2. Generalization Across Independent Datasets

To further evaluate the stability and transferability of the proposed KDH-Net architecture, additional experiments were conducted on four independent kidney CT datasets [36,37,38,39] with varying data distributions, acquisition settings, and class imbalance characteristics. These experiments are intended to assess cross-dataset generalization behavior, while the primary evaluation of this study is performed under a patient-level protocol to ensure strict data separation and avoid data leakage.

It is important to note that evaluation protocols may differ across datasets depending on their structure and annotation format. In particular, some publicly available datasets are organized at the image level without explicit patient grouping, which may allow images from the same subject to appear across training and test sets. In contrast, the primary dataset in this study is strictly partitioned at the patient level, ensuring that all images from a given subject are confined to a single subset. This eliminates potential data leakage and provides a more realistic assessment of model generalization to unseen patients.

As summarized in Table 11, KDH-Net demonstrates consistently strong performance across external datasets, achieving accuracy values ranging from 0.96 to 0.99 despite variations in data distribution and acquisition conditions. The relatively narrow confidence intervals indicate stable predictions, while the gradual increase in standard error across datasets reflects expected sensitivity to domain shifts.

For the primary dataset evaluated under the patient-level protocol, the model achieves an accuracy of 0.93 with a macro F1-score of 0.91, providing a more realistic assessment of generalization performance. Compared to external datasets, this difference reflects the stricter evaluation setting imposed by patient-wise separation, which prevents any overlap of subject-specific features between training and testing and ensures evaluation on entirely unseen patients. Therefore, performance differences across datasets should be interpreted in the context of their respective evaluation protocols rather than as direct comparisons.

Agreement and calibration metrics further support these findings. As shown in Table 12, the model maintains promising agreement across datasets, with Cohen’s *κ* and MCC values above 0.94 for external datasets and approximately 0.80 for the primary dataset. The higher expected calibration error (ECE = 0.16) observed in the primary dataset reflects the increased difficulty of patient-level evaluation, while remaining within an acceptable range for reliable probabilistic prediction.

### 5.3. Clinical Implications and Deployment Readiness

The results demonstrate that KDH-Net provides reliable predictions under a patient-level evaluation setting, which better reflects real clinical deployment conditions. The model exhibits stable confidence estimates, indicating that predicted probabilities are meaningfully aligned with actual outcomes and can support decision-making. Explainability through Grad-CAM further enhances model transparency by highlighting anatomically relevant regions associated with each prediction. These visualizations enable clinicians to qualitatively verify whether the model focuses on plausible renal structures, supporting interpretability without disrupting the diagnostic workflow. Importantly, the proposed system is designed as a clinical decision support tool rather than an autonomous diagnostic system. By combining calibrated predictions with interpretable visual explanations, KDH-Net can assist radiologists in prioritization and secondary review, aligning with human-in-the-loop clinical practice.

### 5.4. Limitations and Future Work

Despite the promising results, several limitations should be noted. First, class imbalance remains inherent in kidney CT datasets, particularly for Stone and Tumor classes, which may affect performance in rare-case scenarios. Second, the framework is evaluated using CT images only; incorporating additional clinical information could further improve stability. Although multiple datasets were used, the primary evaluation was conducted under a patient-level protocol, and broader multi-center validation with prospectively collected clinical data is still required to fully assess generalizability. Furthermore, the explainability analysis relies on Grad-CAM without expert-annotated ground truth, limiting quantitative validation of attention maps. Future work will focus on multimodal integration, large-scale clinical validation, and incorporation of expert annotations to enhance interpretability and real-world applicability.

## 6. Conclusions

This study presents KDH-Net, a hybrid deep learning framework for multiclass kidney disease classification from CT images, integrating EfficientNetB0, ResNet50, and MobileNetV2 through feature-level fusion. The model demonstrates promising performance under a patient-level evaluation protocol, providing a more realistic assessment of generalization to unseen subjects. Beyond classification accuracy, the framework emphasizes reliability and interpretability. Confidence analysis indicates meaningful alignment between prediction confidence and correctness, while Grad-CAM visualizations highlight anatomically relevant regions, supporting transparent and clinically interpretable decision-making. Experimental results across multiple datasets further demonstrate the stability and transferability of the proposed architecture under varying data conditions. Overall, KDH-Net provides a reliable and practical foundation for clinical decision support in kidney disease assessment.

## Figures and Tables

**Table 1:** Literature Summary of Kidney CT Studies.

Study	Dataset	Model	Classes	Performance	XAI	Calibration	Key Limitation
[4]	KAUH	CNN-6, ResNet50	2	CNN-6: 97%; ResNet50: 96%	No	No	Evaluation limited to a single institutional dataset.
[10]	IQ-OTH/NCCD and CT Kidney dataset	Xception	4	Acc = 99.39%	No	No	Limited slice context due to 2D model.
[28]	CT kidney lesion images	IED-ResUNet, HCNN	11	99.60%	No	No	Not validated on diverse external datasets.
[29]	C4KC-KiTS	IED-ResUNet, HCNN	11	AUC = 0.97	No	No	Not multiclass.
[30]	Public CT	DenseNet	4	Acc = 0.95	Grad-CAM	No	Single dataset.
[31]	Public kidney CT	CNN	4	Acc = 0.91	No	No	Class imbalance.
[32]	Multi-source CT	CNN	3	Acc = 0.94	Grad-CAM	No	No calibration.
[33]	Hospital CT	ResNet	4	Acc = 0.96	Grad-CAM	No	Limited generalization.
[34]	Public CT	Hybrid CNN	4	Acc = 0.97	Grad-CAM	No	Single dataset.

**Table 2:** Structure of the CT Kidney dataset organized at patient (group) level. Each group corresponds to one patient folder containing multiple CT slices.

Class	Patient Groups	Total Images
Normal	48	5077
Cyst	81	3708
Tumor	25	2280
Stone	63	1376
Total	217	12,441

**Table 3:** Data Augmentation Configuration.

Augmentation Type	Parameter Value
Rotation Range	±15°
Width Shift	0.1 (10%)
Height Shift	0.1 (10%)
Zoom Range	0.1–0.2
Horizontal Flip	Enabled
Vertical Flip	Disabled
Fill Mode	Nearest

**Table 4:** Two-Stage Training Strategy and Optimization Configuration.

Category	Stage 1: Frozen Backbone Training	Stage 2: Fine-Tuning
Training objective	Task-specific decision learning	Domain-specific feature adaptation
Trainable parameters	Classification head only	Classification head and final backbone layers
Backbone networks	Fully frozen EfficientNetB0, ResNet50, MobileNetV2	Partially unfrozen EfficientNetB0, ResNet50, MobileNetV2
Layer update scope	No backbone updates	Last *L* layers of each backbone
Batch normalization	Frozen	Frozen
Optimizer	Adam	Adam
Learning rate	1 × 10^−3^	1 × 10^−5^
Loss function	Weighted categorical cross entropy	Weighted categorical cross entropy
Class weighting	Enabled for all classes	Enabled for all classes
Regularization	Dropout in classification head	Dropout in classification head
Optimization constraint	Stable convergence	Controlled parameter refinement
Risk mitigation	Prevents catastrophic forgetting	Reduces overfitting and instability

**Table 5:** Experimental Setup and Training Environment.

Component	Specification
Processor	Intel Core i7-12700H CPU @ 2.30 GHz
GPU	NVIDIA GeForce RTX 3060 Laptop GPU
GPU Memory	6 GB
RAM	16 GB
Framework	TensorFlow 2.10
Programming Language	Python 3.9
Operating System	Windows 10 (64-bit)
Batch Size	32
Epochs	40
Optimizer	Adam
Learning Rate	1 × 10^−3^ (Stage 1), 1 × 10^−5^ (Stage 2)

**Table 6:** Class-wise and Overall Performance of KDH-Net.

Class	Precision	Recall	F1-Score		Support
Cyst	0.95	0.96		0.95	618
Normal	0.96	0.91		0.93	760
Stone	0.89	0.95		0.92	218
Tumor	0.82	0.86		0.84	323
Overall Accuracy			0.93		
Macro Avg	0.91	0.92		0.91	
Weighted Avg	0.93	0.93		0.93	

**Table 7:** Bootstrap-based statistical evaluation of model performance (*n* = 1000).

Metric	Value
Mean Accuracy	0.8570
95% Confidence Interval	[0.842, 0.872]
99% Confidence Interval	[0.837, 0.877]
Standard Error	0.0002

**Table 8:** Advanced evaluation metrics and statistical interpretation.

Category	Metric	Value	Interpretation
Error-based	Hamming Loss	0.1433	Moderate misclassification rate
	Zero-One Loss	0.1433	Consistent prediction errors
	Log Loss	0.4828	Stable probabilistic predictions
Overlap-based	Jaccard Score (Macro)	0.7376	Good class-level agreement
	Jaccard Score (Weighted)	0.7561	Balanced performance across classes
Classification	Balanced Accuracy	0.8496	Stable performance under class imbalance
	Matthews Corr. Coef.	0.7964	Promising overall classification quality
Agreement	Cohen’s Kappa	0.7959	Good agreement beyond chance
Calibration	Overall ECE	0.1568	Moderate calibration error
	Confidence Gap	0.1055	Meaningful confidence separation

**Table 9:** Quantitative evaluation of Grad-CAM explanations using perturbation-based metrics.

Metric	Value
Confidence Drop	0.2913
Confidence on Masked Region	0.4639
Deletion Score	0.5936
Insertion Score	0.4247

**Table 10:** Comparative performance of KDH-Net and baseline CNN architectures using patient-level evaluation.

Model	Acc	Macro F1	W-Prec	W-Rec	W-F1	Cyst F1	Normal F1	Stone F1	Tumor F1
EfficientNetB0	0.1136	0.0510	0.0129	0.1136	0.0232	0.0000	0.0000	0.2040	0.0000
ResNet50	0.7196	0.7063	0.7963	0.7196	0.7191	0.8346	0.6885	0.7594	0.5426
MobileNetV2	0.6373	0.5500	0.6692	0.6373	0.6215	0.6565	0.7804	0.5008	0.2624
VGG16	0.1146	0.0820	0.1089	0.1146	0.0739	0.1757	0.0000	0.1524	0.0000
InceptionV3	0.8004	0.7898	0.8589	0.8004	0.8087	0.8658	0.8178	0.7753	0.7005
DenseNet121	0.8828	0.8495	0.8956	0.8828	0.8782	0.9068	0.9312	0.8258	0.7342
Xception	0.8067	0.7670	0.8027	0.8067	0.7997	0.9359	0.8102	0.8245	0.4974
NASNetMobile	0.4237	0.2446	0.3519	0.4237	0.2799	0.0000	0.5817	0.3154	0.0812
ResNet50 + MobileNetV2	0.8791	0.8528	0.8752	0.8791	0.8737	0.9062	0.9236	0.9148	0.6667
ResNet50 + EfficientNetB0	0.8140	0.7746	0.8415	0.8140	0.8136	0.8149	0.9032	0.7100	0.6703
MobileNetV2 + EfficientNetB0	0.8280	0.8012	0.8898	0.8280	0.8367	0.7355	0.9588	0.6233	0.8872
KDH-Net (Proposed)	0.93	0.91	0.93	0.93	0.93	0.95	0.93	0.92	0.84

**Table 11:** Generalization performance of KDH-Net across independent datasets.

Dataset	Total Images	Train/Val/Test	Accuracy	Macro F1	Weighted F1	95% CI	Std. Error
Dataset A	9564	7669/947/948	0.98	0.98	0.98	[0.97, 0.98]	0.0044
Dataset B	9555	6674/1923/958	0.97	0.97	0.97	[0.96, 0.98]	0.0051
Dataset C	15,102	13,200/946/956	0.96	0.98	0.98	[0.95, 0.97]	0.0061
Dataset D	12,446	8712/1867/1867	0.99	0.99	0.99	[0.99, 0.99]	0.0018
Primary	12,441	8765/1757/1919	0.93	0.91	0.93	[0.84, 0.87]	0.0002

**Table 12:** Agreement and calibration metrics across datasets.

Dataset	Cohen’s *κ*	MCC	Mean ECE
Dataset A	0.97	0.97	0.01
Dataset B	0.96	0.96	0.01
Dataset C	0.94	0.94	0.02
Dataset D	0.99	0.99	0.03
Primary	0.80	0.80	0.16

## Data Availability

The data presented in this study are openly available in Kaggle at https://www.kaggle.com/datasets/hadighahroudi/ct-kidney-dataset-normal-cyst-tumor-and-stone/data, accessed on 13 March 2022.
